# Socioeconomic Status and Overall Survival Among Patients With Hematological Malignant Neoplasms

**DOI:** 10.1001/jamanetworkopen.2024.1112

**Published:** 2024-03-04

**Authors:** Lars Hernández Nielsen, Daniel Tuyet Kristensen, Lasse Hjort Jakobsen, Martin Bøgsted, Henrik Gregersen, Jakob Madsen, Marianne Tang Severinsen, Rasmus Froberg Brøndum

**Affiliations:** 1 Center for Clinical Data Science, Department of Clinical Medicine, Aalborg University and Research, Education and Innovation, Aalborg University Hospital, Aalborg, Denmark; 2 Department of Hematology, Clinical Cancer Research Center, Aalborg University Hospital, Aalborg, Denmark; 3 Department of Clinical Medicine, Aalborg University, Aalborg, Denmark; 4 Department of Mathematical Sciences, Aalborg University, Aalborg, Denmark

## Abstract

**Importance:**

In recent years, there has been a focus on reducing the socioeconomic gap in survival for hematological malignant neoplasms. Understanding recent developments is important to develop further intervention to improve care.

**Objective:**

To investigate the temporal trend in associations of socioeconomic status (SES) with survival among 3 aggressive hematological malignant neoplasms: multiple myeloma (MM), acute myeloid leukemia (AML), and diffuse large B-cell lymphoma (DLBCL).

**Design, Setting, and Participants:**

This nationwide, population-based cohort study used retrospectively collected data from 3 clinical registries of patients diagnosed in Denmark between January 1, 2005, and December 31, 2020, with follow-up until December 31, 2021. Analyses were stratified by diagnosis year (2005-2009, 2010-2014, and 2015-2020). Participants were patients aged 25 to 65 years with hematological malignant neoplasms. Patients with missing data on education were excluded. Data were analyzed from October 14, 2022, to January 2, 2024.

**Exposure:**

Education was used as a proxy for SES and defined low- and high-SES groups based on the completion of tertiary education.

**Main Outcomes and Measures:**

The main outcome was overall survival (OS), analyzed using Kaplan-Meier (log rank) method and Cox proportional hazards regression adjusted for age, sex, performance status, comorbidities, and disease-specific prognostic indices. Two-year OS through time and survival difference were estimated using flexible parametric survival models.

**Results:**

A total of 5677 patients (median [IQR] age, 58 [51-62] years; 3177 [57.0%] male) were assessed, including 1826 patients with MM, 1236 patients with AML, and 2509 patients with DLBCL. The 2-year OS increased over time for patients with MM (78.8% [95% CI, 75.4%-82.3%] to 91.4% [95% CI, 89.3%-93.5%]), AML (42.2% [95% CI, 37.8%-47.1%] to 52.7% [95% CI, 48.0%-57.9%]), and DLBCL (80.1% [95% CI, 77.4%-82.8%] to 88.1% [95% CI, 86.0%-90.3%]). For MM and DLBCL, no association of SES with survival was observed after adjustment (MM: hazard ratio [HR], 0.99 [95% CI, 0.85-1.15]; DLBCL: HR, 1.08 [95% CI, 0.91-1.29]). For AML, a negative association was observed between low SES and survival (HR, 1.49 [95% CI, 1.25-1.76]), but the association was attenuated in recent years. The difference in hazard for patients with low SES and AML was observed in the first 2 years after diagnosis.

**Conclusions and Relevance:**

These findings suggest that survival has improved among patients with these hematological malignant neoplasms. While patients with MM and DLBCL had increased survival in all groups, disparities were observed in AML outcomes, primarily in the first years after diagnosis. These results suggest that differences originate in factors specific to AML.

## Key Points

**Question:**

Has the socioeconomic gap in survival for patients with hematological malignant neoplasms changed in recent years?

**Findings:**

This cohort study including 5677 patients with hematological malignant neoplasms in Denmark found no significant differences in survival according to socioeconomic status within patients with multiple myeloma, but there were differences for patients with acute myeloid leukemia or diffuse large B-cell lymphoma. The association of socioeconomic status with survival for patients with acute myeloid leukemia was observed mainly during the first 2 years after diagnosis.

**Meaning:**

These findings suggests that there is a need for a continued focus on these patients to reduce inequality in survival.

## Teaser

This cohort study investigates the temporal trends in associations of socioeconomic status with survival among patients with aggressive hematological malignant neoplasms in Denmark.

## Introduction

Socioeconomic status (SES), often measured by educational attainment or income level, is known to impact the course and outcome of cancers.^1^ In countries with tax-funded health care schemes, outcome differences associated with nonbiological factors, such as SES, could indicate unintended barriers in access to health care services. Understanding the mechanisms behind differences in outcome is important to reduce inequality and because differences might reveal opportunities for improvement that could benefit all patients.

There are many possible mechanisms behind inferior outcomes in patients with low SES, and differences likely arise from a mix of factors. Suspected factors include delayed diagnostics, low participation in screening programs, lack of treatment adherence, lower treatment intensity, reduced trial participation, presence of comorbidities, and negative lifestyle factors.^2,3,4,5^ A 2022 meta-analysis of SES inequality in cancer in Nordic countries showed differences in the stage at diagnosis, treatment, and survival across SES groups.^6^ It is especially problematic if differences arise from a lack of access to treatment, which should not be present in countries that, in theory, provide equal access to health care.

Studies on hematological malignant neoplasms have reported inferior survival among patients with low SES compared with patients with high SES. This association has been observed in multiple myeloma (MM), acute myeloid leukemia (AML), and non-Hodgkin lymphoma.^5,7,8^ In general, survival measured across all patient groups has increased over time, but studies have indicated that new treatments have only benefitted patients with high SES, suggesting an increasing social inequality in cancer treatment.^5,7^ However, the association of SES with hematological malignant neoplasms outcomes, in light of past years’ improvements in treatment, has not been fully investigated, and past differences might not persist due to increased focus on reducing social inequalities.

The aim of this study was to compare survival across SES groups for 3 common and aggressive hematological malignant neoplasms, MM, AML, and diffuse large B-cell lymphoma (DLBCL), in adult patients aged 25 to 65 years in Denmark. The analyses were stratified by calendar year to investigate how differences have evolved.

## Methods

In accordance with Danish regulations, this cohort study is registered in an internal project database in the North Denmark Region, and being a register-based study with no patient contact, does not require informed consent or approval from an ethics committee per the European Union general data protection regulation (Art. 6. 1 [e]). We followed the Strengthening the Reporting of Observational Studies in Epidemiology (STROBE) reporting guideline for cohort studies.

### Study Design and Setting

The patients in this Danish nationwide population-based cohort study were identified through 3 disease-specific clinical registries: patients with MM were identified through the Danish National Multiple Myeloma Registry (DMMR); patients with AML, excluding acute promyelocytic leukemia, were identified through the Danish National Acute Leukemia Registry (DNLR); and patients with DLBCL were identified through the Danish National Lymphoma Registry (LYFO). The databases contain an almost complete registration of all patients with these diagnoses.^9,10,11^

### Study Population

Patients had to fulfill the following inclusion criteria: aged between 25 and 65 years at diagnosis, which allows for a completed educational degree and exclusion of patients outside working age to reduce noise from increased mortality due to age; diagnosed with MM, AML, or DLBCL between January 1, 2005, and December 31, 2020; and available information on educational level and follow-up. Missing data on education were most likely due to immigration.

### Clinical Information and Definitions

Information on the date of diagnosis, World Health Organization (WHO) performance score, Charlson Comorbidity Index (CCI), and disease-specific prognostic variables were collected from clinical registries. The disease-specific prognoses were assessed using disease-specific indices. MM was assessed using International Staging System for Multiple Myeloma^12^ (ISS), with prognosis categorized as favorable (stage I), intermediate (stage II), or adverse (stage III). AML was assessed with Grimwade cytogenetic classification,^13^ categorized as favorable, intermediate, and adverse. DLBCL was assessed using the International Prognostic Index for DLBCL^14^ (IPI), categorized as favorable (0-1), intermediate (2-3), and adverse (4-5).

Data on sex, date of birth, date of emigration, and date of death were obtained from the Danish Central Person Register. Data on highest attained education level were obtained from the Danish educational registries.^15^ As a crude proxy for SES, educational level was divided into low SES and high SES groups based on the completion of tertiary education. A sensitivity analysis using income as a proxy for SES was also included. Further details about definitions are provided in the eMethods in Supplement 1.

### Statistical Analysis

Patients were followed-up from the date of diagnosis until death, emigration, or end of follow-up (December 31, 2021). Overall survival (OS) was estimated using the Kaplan-Meier method, and differences were tested using the log-rank test. Hazard ratios (HRs) were estimated using Cox proportional hazards regression with the high SES strata as reference. This was performed for both univariable and multivariable models. We estimated a confounder-adjusted model, including sex and age (continuous), and a mediator-adjusted model, including sex, age (continuous), CCI, WHO performance score, and disease-specific prognostic index. We distinguish between confounding and mediating factors because some factors are associated with SES (eg, comorbidity or performance score) and thus the association of SES with OS could be mediated through these factors.

Analyses were stratified by calendar period at diagnosis. The periods were defined as 2005 to 2009, 2010 to 2014, and 2015 to 2020. The proportional hazards assumption was tested by visually inspecting the hazards as a function of time.

We estimated the 2-year OS using a flexible parametric survival model containing a time-varying coefficient for year of diagnosis. This was used to model temporal changes in 2-year OS. In this model, the baseline hazard was modeled using natural cubic splines with knots in the quantiles. We modeled the 2 subpopulations (high vs low SES) separately and obtained estimates for each group across time, along with 95% pointwise CIs. We plotted smooth-in-time survival difference from a model containing SES as a covariate, and natural cubic splines were used in the modeling of the baseline hazard. The models, which are dependent on modeling the smooth hazard function, were checked for sensitivity to different numbers of spline knots.

When comparing HRs across periods, differences in length of follow-up might influence results. This can be problematic if the proportional hazard assumption is broken, for example, if the HR is larger close to treatment.^16^ To account for this, we performed sensitivity analyses restricting follow-up to 6 years across all 3 periods. We also included a post hoc supplemental analysis comparing treatment patterns among patients with AML to investigate the association between SES and the rates of intensive chemotherapy and allogeneic stem cell transplantation.

Statistical significance was determined at *P* < .05. Data management and statistical analysis were performed on servers at Statistics Denmark using R statistical software version 4.0.3 (R Project for Statistical Computing) and R packages survminer,^17^ rstpm2,^18^ and forestploter.^19^ Data were analyzed from October 14, 2022, to January 2, 2024.

## Results

### Patient Characteristics

**Figure 1.**
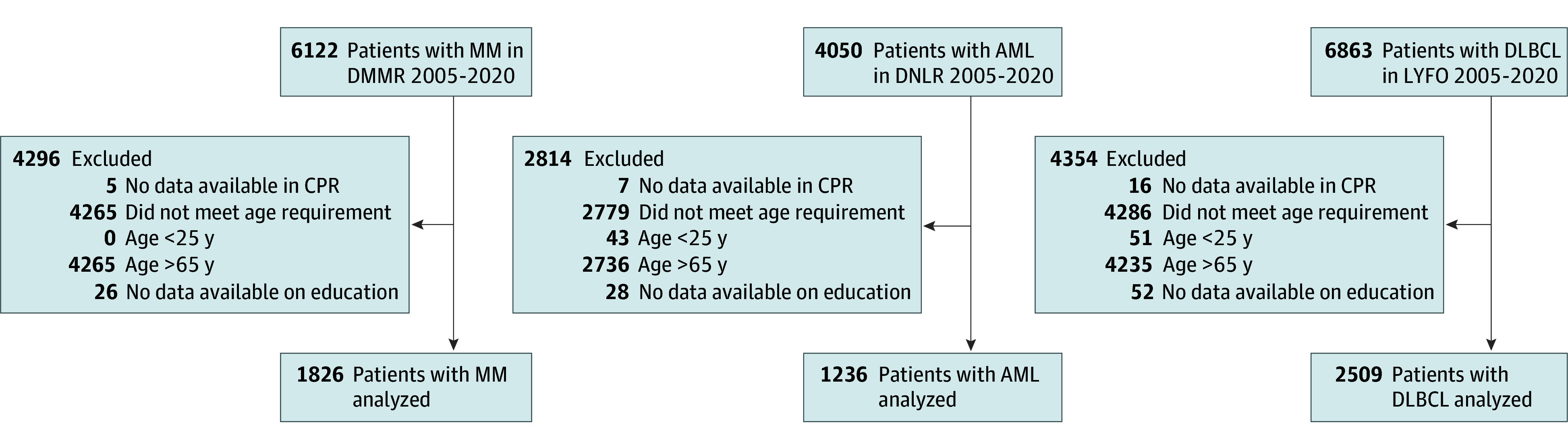
Flowchart of Study Exclusion Criteria The study includes patients with multiple myeloma (MM) from the Danish National Multiple Myeloma Registry (DMMR), patients with acute myeloid leukemia (AML) from the Danish National Acute Leukemia Registry (DNLR), and patients with diffuse large B-cell lymphoma (DLBCL) from the Danish National Lymphoma Registry (LYFO). CPR indicates Central Person Register.

A total of 17 035 patients with MM, AML, or DLBCL were identified in DMMR, DNLR, and LYFO, respectively. Among these, 11 464 patients were excluded: 11 350 patients (99.1%) did not fall within the age range, and 134 patients (0.9%) had missing information on educational level or follow-up (Figure 1). The final cohort included 5677 patients (median [IQR] age, 58 [51-62] years; 3177 [57.0%] male), with 1826 patients (1032 [56.5%] male; median [IQR] age, 59 [53 to 63] years) in the MM cohort, 1236 patients (645 [52.2%] male; median [IQR] age, 56 [47 to 61] years) in the AML cohort, and 2509 patients (1500 [59.8%] male; median [IQR] age, 57 [49 to 62] years) in the DLBCL cohort. Baseline characteristics for the 3 disease groups stratified by SES are shown in the Table. Across disease groups, the high SES group had a higher proportion of females, lower age, and lower CCI compared with the low SES group (Table). Furthermore, WHO performance status was lower in the high SES group compared with the low SES group (Table). The proportion of patients categorized as high SES increased over time in all disease groups.

**Table.** Patient Characteristics by SES

Characteristic	MM			AML			DLBCL		
	Patients, No. (%)		*P* value^a^	Patients, No. (%)		*P* value^a^	Patients, No. (%)		*P* value^a^
	Low SES (n = 1263)	High SES (n = 563)		Low SES (n = 898)	High SES (n = 338)		Low SES (n = 1772)	High SES (n = 737)	
Sex									
Female	518 (41.0)	276 (49.0)	.002	393 (43.8)	198 (58.6)	<.001	683 (38.5)	326 (44.2)	.009
Male	745 (59.0)	287 (51.0)		505 (56.2)	140 (41.4)		1089 (61.5)	411 (55.8)	
Age, y									
Median (IQR)	59 (54-63)	59 (53-62)		57 (48-62)	55 (45-60)		58 (50-62)	55 (46-61)	
25-39 y	18 (1.4)	18 (3.2)	.03	105 (11.7)	56 (16.6)	.007	134 (7.6)	108 (14.7)	<.001
40-54 y	339 (26.8)	154 (27.4)		261 (29.1)	113 (33.4)		536 (30.2)	243 (33.0)	
55-65 y	906 (71.7)	391 (69.4)		532 (59.2)	169 (50.0)		1102 (62.2)	386 (52.4)	
WHO-PS									
0	526 (41.6)	291 (51.7)	<.001	394 (43.9)	172 (50.9)	.05	999 (56.4)	480 (65.1)	<.001
1	441 (34.9)	168 (29.8)		358 (39.9)	125 (37.0)		493 (27.8)	188 (25.5)	
≥2	282 (22.3)	101 (17.9)		146 (16.3)	41 (12.1)		273 (15.4)	69 (9.4)	
Immigration status									
Danish	1175 (93.0)	500 (88.8)	.003	835 (93.0)	305 (90.2)	.14	1626 (91.8)	648 (87.9)	.004
Non-Danish	88 (7.0)	63 (11.2)		63 (7.0)	33 (9.8)		146 (8.2)	89 (12.1)	
Disease-specific prognostic index^b^									
Favorable	421 (33.3)	191 (33.9)	.99	61 (6.8)	24 (7.1)	.21	710 (40.1)	321 (43.6)	.26
Intermediate	389 (30.8)	172 (30.6)		417 (46.4)	178 (52.7)		798 (45.0)	327 (44.4)	
Adverse	289 (22.9)	130 (23.1)		190 (21.2)	59 (17.5)		189 (10.7)	63 (8.5)	
Missing	164 (13.0)	70 (12.4)		230 (25.6)	77 (22.8)		75 (4.2)	26 (3.5)	
Charlson Comorbidity Index									
0	794 (62.9)	395 (70.2)	.02	609 (67.8)	279 (82.5)	<.001	1095 (61.8)	562 (76.3)	<.001
1	248 (19.6)	85 (15.1)		196 (21.8)	41 (12.1)		396 (22.3)	119 (16.1)	
2	143 (11.3)	54 (9.6)		45 (5.0)	11 (3.3)		127 (7.2)	28 (3.8)	
≥3	78 (6.2)	29 (5.2)		48 (5.3)	7 (2.1)		154 (8.7)	28 (3.8)	
Time period									
2005-2009	399 (31.6)	143 (25.4)	.03	336 (37.4)	97 (28.7)	.003	631 (35.6)	212 (28.8)	<.001
2010-2014	373 (29.5)	175 (31.1)		289 (32.2)	107 (31.7)		567 (32.0)	222 (30.1)	
2015-2020	491 (38.9)	245 (43.5)		273 (30.4)	134 (39.6)		574 (32.4)	303 (41.1)	

Abbreviations: AML, acute myeloid leukemia; DLBCL, diffuse large B-cell lymphoma; MM, multiple myeloma; SES, socioeconomic status; WHO-PS, World Health Organization performance score.

^a^ *P* values calculated from χ^2^ test between high SES group and low SES group.

^b^ Disease-specific prognostic indices were categorized as: AML, cytogenetic risk category^13^; DLBCL, International Prognostic Index for DLBCL^14^; and MM, International Staging System for Multiple Myeloma.^12^

The median follow-up times (calculated using reverse Kaplan-Meier method) were 7.4 (95% CI, 7.1 to 7.8) years for MM, 8.7 (95% CI, 7.9 to 9.3) years for AML, and 9.2 (95% CI, 8.8 to 9.5) years for DLBCL. The 2-year OS increased for MM, from 78.8% (95% CI, 75.4% to 82.%3) to 91.4% (95% CI, 89.3% to 93.5%); AML, from 42.2% (95% CI, 37.8% to 47.1%) to 52.7% (95% CI, 48.0% to 57.9%); and DLBCL, from 80.1% (95% CI, 77.4% to 82.8%) to 88.1% (95% CI, 86.0% to 90.3%) from the first period (2005 to 2009) to the last period (2015 to 2020).

### Survival by Socioeconomic Status

**Figure 2.**
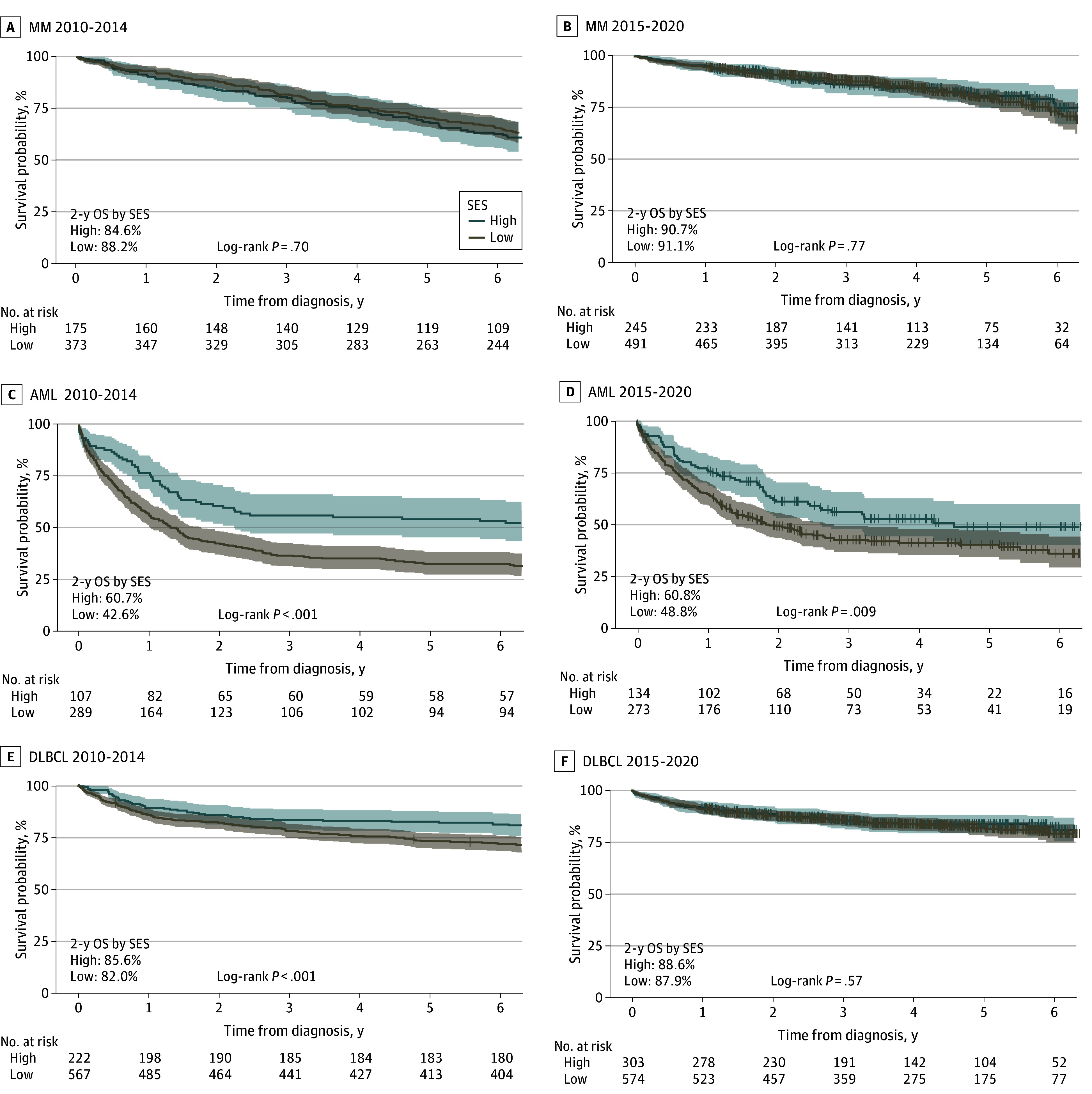
Overall Survival (OS) Probability by Socioeconomic Status (SES) and Year of Diagnosis AML indicates acute myeloid leukemia; DLBCL, diffuse large B-cell lymphoma; and MM, multiple myeloma. *P* values are from log-rank tests. Censoring is indicated by vertical line. Shaded areas indicate 95% CIs.

Kaplan-Meier plots for the 3 diagnoses are presented in Figure 2 for the 2010 to 2014 and 2015 to 2020 periods. The Kaplan-Meier plots for 2005 to 2009 period and full 2005 to 2020 period are presented in eFigure 1 in Supplement 1. For patients with MM, no significant difference was found between the low and high SES groups using log-rank tests across the 3 periods (Figure 2A; eFigure 1 in Supplement 1). For AML, significant differences were found in all 3 periods (Figure 2B; eFigure 1 in Supplement 1). For DLBCL, significant differences were found for 2005 to 2009 and 2010 to 2014, but no significant difference was seen in the 2015 to 2020 period (Figure 2C; eFigure 1 in Supplement 1). For the whole 2005 to 2020 period differences in AML and DLBCL were statistically significant.

**Figure 3.**
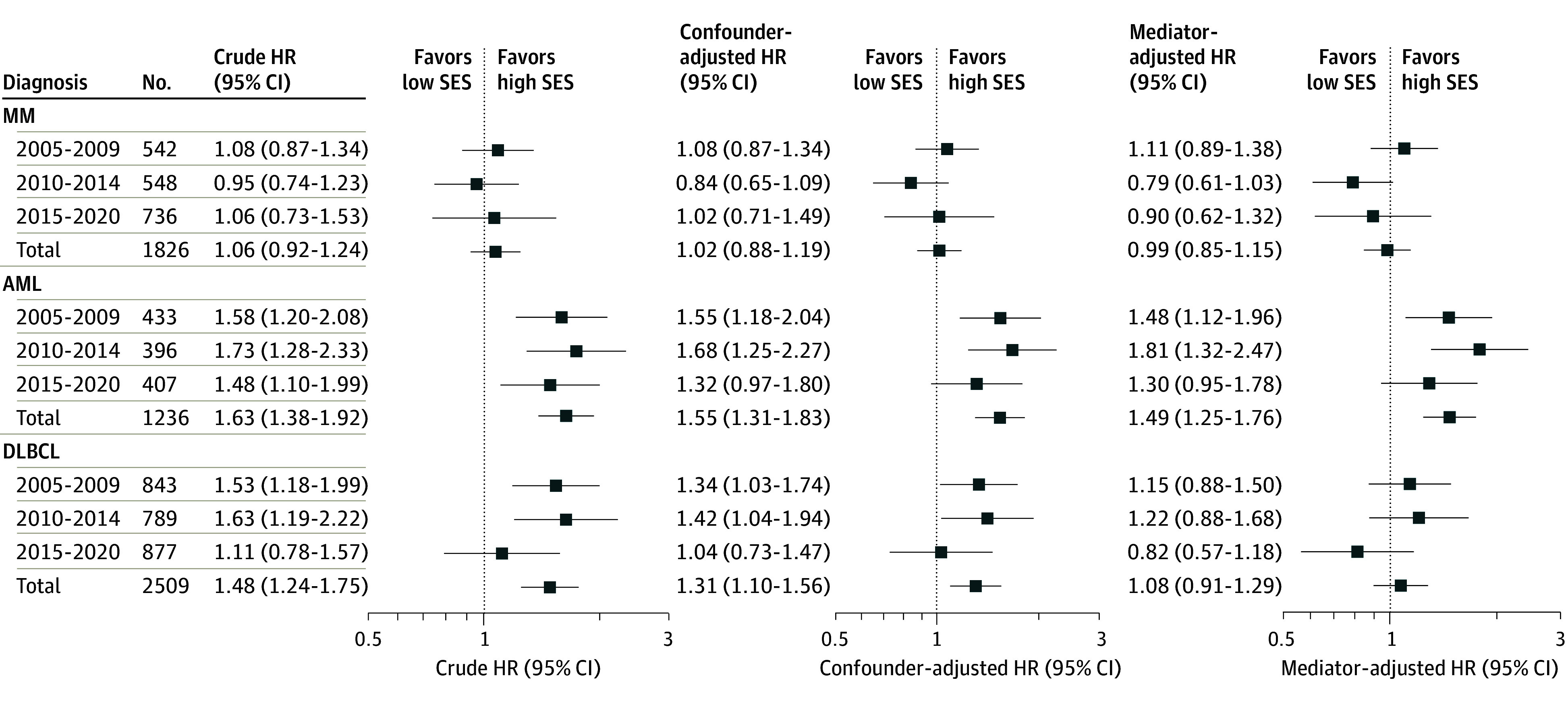
Estimated Hazard Ratios (HRs) From Univariable and Multivariable Cox Regressions for Socioeconomic Differences HRs are estimated using patients with high socioeconomic status (SES) as reference. The confounder-adjusted model includes age and sex, and the mediator-adjusted model includes age, sex, performance score, comorbidity, and disease-specific prognostic index. AML indicates acute myeloid leukemia; DLBCL, diffuse large B-cell lymphoma; and MM, multiple myeloma.

The results from univariable and multivariable Cox regression analyses investigating the association of SES with OS adjusted for mediators and confounders are shown in Figure 3. For MM, the confounder-adjusted model found no significant difference overall (2005 to 2020: HR, 1.02 [95% CI, 0.88 to 1.19]) or for the individual periods (Figure 3). The results for the mediator-adjusted model were similar. For AML, the confounder-adjusted model found higher hazard for the low SES group overall (2005 to 2020: HR, 1.55 [95% CI, 1.31 to 1.83]), although there was no significant difference for the 2015 to 2020 period (Figure 3). Adjusting for mediators reduced the difference, although patients with low SES still had significantly higher hazard (HR, 1.49 [95% CI, 1.25 to 1.76]). For DLBCL, the confounder-adjusted model from 2005 to 2020 showed an increased hazard for patients with low SES (HR, 1.31 [95% CI, 1.10 to 1.56]), with period-specific differences seen for the 2 earliest periods, whereas this was not seen for the most recent period (Figure 3). In the mediator-adjusted model, the association of SES with OS decreased, resulting in no significant differences in any periods or for 2005 to 2020 overall (HR, 1.08 [95% CI, 0.91 to 1.29]) (Figure 3).

Restricting to a maximum follow-up of 6 years, we found similar results to the unrestricted univariate and multivariable Cox regressions (eFigure 2 in Supplement 1). We also investigated the effect of using income as a proxy for SES, and results showed a constant small difference across all groups (eFigures 3-6 and eTable in Supplement 1).

### Smooth Survival Differences

**Figure 4.**
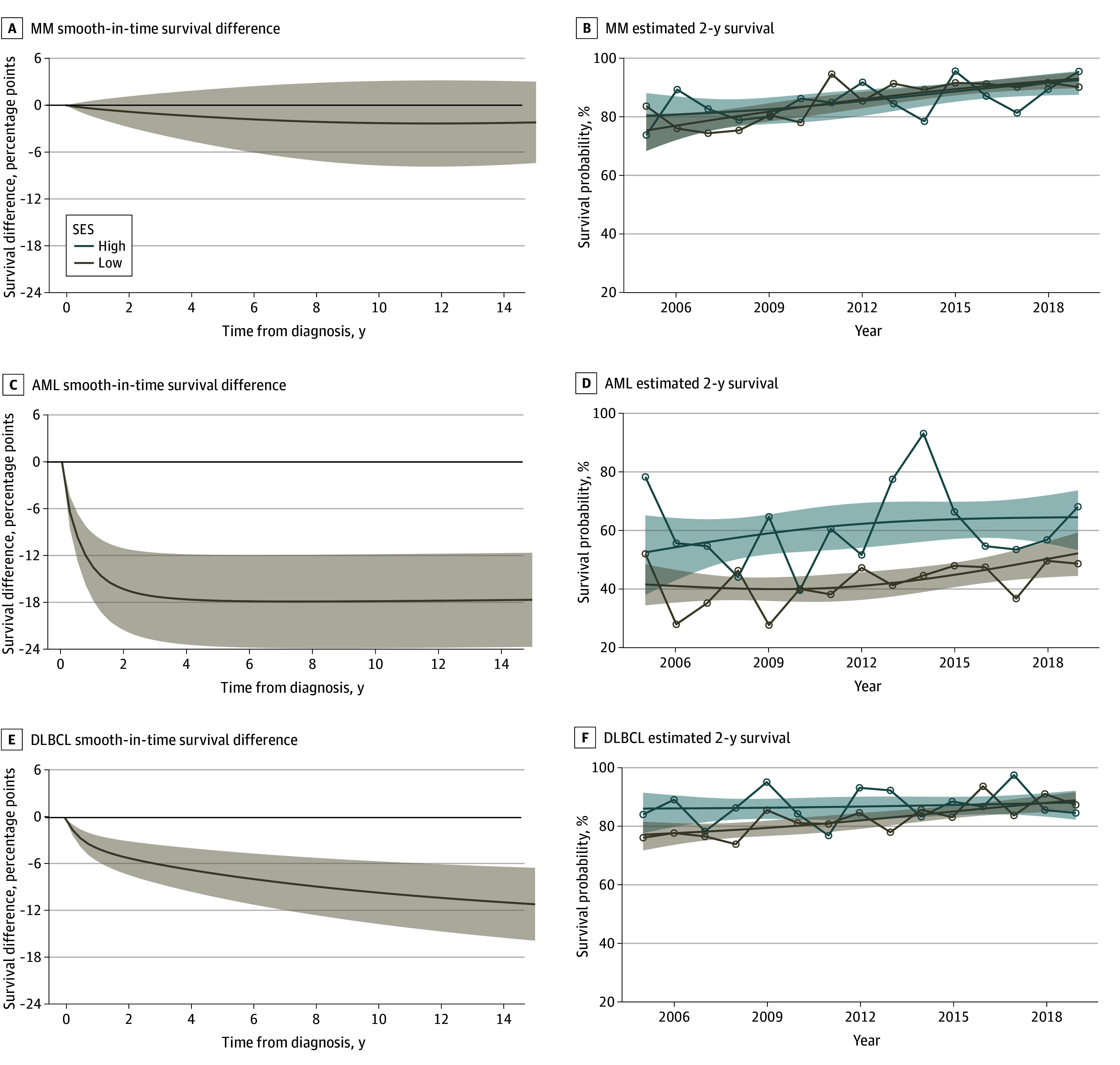
Estimated Socioeconomic Differences Using Flexible Parametric Survival Models AML indicates acute myeloid leukemia; DLBCL, diffuse large B-cell lymphoma; and MM, multiple myeloma. Shaded areas indicate 95% CIs.

The smooth-in-time survival difference across the entire period for the 3 diagnoses are displayed in Figure 4A through C. There were significant no differences in survival between the high and low SES strata for MM. The estimated difference for low SES compared with high SES was −0.8 (95% CI, −2.8 to 1.1) percentage points at 2 years and −1.6 (95% CI, −5.4 to 2.1) percentage points at 5 years. In contrast, for AML, survival diverged in the first 2 to 3 years after diagnosis and was comparable thereafter (Figure 4B). We estimated the difference for low SES vs high SES at −16.3 (95% CI, −21.5 to −11.1) percentage points at 2 years and −17.7 (95% CI, −23.6 to −11.9) percentage points at 5 years. For DLBCL, the difference in survival between patients with low vs high SES slowly increased. At 2 years, this difference was −5.1 (95% CI, −7.3 to −3.0) percentage points, and at 5 years, it was −7.3 (95% CI, −10.3 to −4.3) percentage points.

### Estimated 2-Year Survival by Year of Diagnosis

The smoothed estimated 2-year survival rates from 2005 to 2019 from the flexible parametric survival models are shown in Figure 4D through F. For MM, the risk ratio was very similar through time, whereas for AML, a large difference was observed. Notably, in 2013 and 2014, the high SES group experienced low mortality (Figure 4E). For DLBCL, the difference gradually diminished from 2005 to 2019. As smoothing is highly dependent on the number of spline knots when modeling the baseline hazard, we included smoothed estimated 2-year survival with a varying number of spline knot points (eFigure 7 in Supplement 1).

### Treatment Type in AML

For AML, treatment is normally distinguished between intensive and nonintensive induction chemotherapy. In the AML cohort, 96.8% of patients with high SES received intensive treatment, while 93.6% of patients in the low SES group received intensive therapy (χ^2^_1_ = 4.02; *P* = .045). The rate of allogeneic transplantations for patients with high SES was 35.2%, while for patients with low SES, it was 25.8% (χ^2^_1_ = 9.44; *P* = .002). Including treatment intensity and transplantation status reduced the HRs in the confounder-adjusted model for AML for 2005-2020 (HR, 1.48 [95% CI, 1.24 to 1.77]).

## Discussion

In this nationwide population-based cohort study, we investigated the association of SES with OS in 3 common hematological malignant neoplasms. Overall, we observed improvements in OS for all 3 diseases during the study period. For patients diagnosed with MM, we found no significant differences in outcome associated with SES. For patients with DLBCL and high SES, we observed better outcomes in the 2 earlier periods compared with patients with low SES. However, this difference was reduced in recent years and disappeared after adjusting for mediators. For AML, we found a significant association of SES with outcome throughout the study period, favoring patients with high SES. This association remained after adjusting for confounders and mediators, particularly in the earlier periods. When income was used as a proxy for SES, we observed small but consistent differences across all diagnoses. We suspect that defining SES by income prior to diagnosis was susceptible to bias to a larger degree than education level due to disease stage, eg, some patients experienced slow onset of symptoms and therefore moved to the low-SES group because they stopped working a few years before diagnosis; however, we did not investigate whether this was the case.

Previous studies have assessed the association between SES and outcome in hematological malignant neoplasms and observed differences. For MM, most studies have found an association between low SES and inferior outcomes.^20,21,22,23,24,25,26^ A Swedish study including patients without age restriction diagnosed with MM between 1973 and 2005 is most comparable with our results.^7^ The study by Kristinsson et al^7^ found that the differences in outcomes between white-collar workers and blue-collar workers increased over time (2000 to 2005: HR, 1.31 [95% CI, 1.07-1.60]), while the difference between low and high SES groups in our earliest period was not statistically significant. This could indicate that changes in clinical practice have reduced inequality or reflect the differing populations.

In DLBCL and more generally non-Hodgkin lymphoma, several studies have indicated SES as a factor associated with outcome. In this context, SES includes educational level^8,26,27,28^ and area-level socioeconomic deprivation,^29,30,31,32^ whereas other studies have found no association between SES and outcome.^31,33^ In a Swedish study including patients with DLBCL diagnosed between 2007 and 2013, longer education was associated with lower lymphoma-specific mortality.^28^

When adjusting for mediators in DLBCL, we found no statistically significant association between SES and survival. This does not imply that inequality is nonexistent within DLBCL, it merely suggests that survival is likely affected through many mediators, such as comorbidity. Therefore, to reduce differences in outcomes, focus should be placed on lifestyle-induced factors.

There was a significant mediator-adjusted association of SES with AML outcomes (HR, 1.49 [95% CI, 1.25-1.76]); however, when subdividing our study period, the difference could in part be attributed to the earlier periods, indicating a reduction in the inequality of outcome. The literature lacks more recent studies for direct comparison, but several previous studies have found a negative association between SES and outcome^5,7,34^

When examining the survival difference in AML, we found that most of the increased hazard for the low SES group was within 2 years after diagnosis. This observation could indicate that differences in outcome are related to the management of treatment-related complications and primary refractory or relapsed disease, since most relapses occur within the first 2 to 3 years after the achievement of complete remission.^35,36^ Consistent with this observation, a 2017 study found that marital status and neighborhood SES are independent risk factors for 60-day mortality in patients with AML.^37^ In addition, the management of common complications related to the treatment of AML, ie, neutropenic infections, where rapid antibiotic management is required,^38^ could also contribute to this difference. Outside factors, such as travel time or distance to specialized care, would affect the time from onset of fever to antibiotic administration. A 2019 Danish study found that patients living furthest from specialized centers generally had lower income and lower educational level. Surprisingly, the distance to specialized centers did not influence the rates of complete remission, intensive chemotherapy treatment, or mortality.^39^ In light of these findings, it seems unlikely that distance to treatment could explain the differences observed across SES in patients with AML.

A California-based study found that low SES was associated with lower rates of induction chemotherapy and allogeneic stem-cell transplantation in patients with AML.^40^ Our study only included patients who could be expected to tolerate intensive treatment (ie, aged 25-65 years), still we observed small differences by SES in the amount of intensive treatment and rate of transplantation for patients with AML. However, these factors could only explain a small part of the difference in HR for the patients with low SES.

A strength of our study is the use of nationwide Danish registries that offer individual-level educational and economic data with high validity and coverage. Furthermore, the use of parametric survival models allowed us to analyze the year-to-year survival across SES without results depending on a specific division of the period.

### Limitations

This study has some limitations. One limitation is the choice of proxy for SES, in our case educational attainment and income. Both are crude and naturally leave out many aspects that might be caught by including other SES proxies. Distance to specialized centers could also affect attitudes toward treatment or participation in clinical trials, as it is well established that patients with cancer with lower income are less likely to participate in clinical trials.^4^ Investigating SES is also complicated by several lifestyle-related prognostic factors entangled with low SES, such as smoking^41^ or increased adiposity, where the prognostic impact is more controversial,^42,43^ but these data are generally not recorded in the Danish registries used in this study.

## Conclusions

In this cohort study, we found no notable associations of SES with outcomes in MM and DLBCL. For AML, we found significant associations in outcome in the earlier periods; however, this association diminished over time. Since this study was conducted in a universal and tax-funded health care system, we believe that the observed inferior outcomes for patients with low SES can be overcome by increased focus and equal access to health care services. Further investigations of the differences are important to improve understanding of what causes the differences in the first years following AML diagnosis, as findings could possibly be used to improve outcomes for all patients.

## Notes

Supplement 1. **eMethods.** Definitions of Variables Used in the Analysis **eFigure 1.** All Periods and Unstratified Kaplan-Meier Curves for Overall Survival **eFigure 2.** Estimated Hazard Ratios From Univariable and Multivariable Cox Regressions for Socioeconomic Differences: Follow-Up Restricted to 6 Years From Diagnosis **eFigure 3.** Unstratified Kaplan-Meier Curves for Overall Survival, Using Income as a Proxy for SES **eFigure 4.** Overall Survival Probability by SES and Year of Diagnosis, Using Income as a Proxy for SES **eFigure 5.** Estimated SES Differences Using Flexible Parametric Survival Models, Using Income as a Proxy for SES **eFigure 6.** Estimated Hazard Ratios From Univariable and Multivariable Cox Regressions for SES Differences, Using Income as a Proxy for SES **eFigure 7.** 2-Year Survival Using 1 and 6 Degrees of Freedom (Spline Knots) in Modeling of the Baseline Hazard **eTable.** Patient Characteristics by SES, Using Income as a Proxy for SES

Supplement 2.
Data Sharing Statement
