# Supplementary material for: Socioeconomic Status and Overall Survival Among Patients With Hematological Malignant Neoplasms
Source: JAMA Netw Open. 2024 Mar 4;7(3):e241112. doi: 10.1001/jamanetworkopen.2024.1112 (PMC10912957; doi:10.1001/jamanetworkopen.2024.1112)
Supplement: Supplement 1. — eMethods. Definitions of Variables Used in the Analysis eFigure 1. All Periods and Unstratified Kaplan-Meier Curves for Overall Survival eFigure 2. Estimated Hazard Ratios From Univariable and Multivariable Cox Regressions for Socioeconomic Differences: Follow-Up Restricted to 6 Years From Diagnosis eFigure 3. Unstratified Kaplan-Meier Curves for Overall Survival, Using Income as a Proxy for SES eFigure 4. Overall Survival Probability by SES and Year of Diagnosis, Using Income as a Proxy for SES eFigure 5. Estimated SES Differences Using Flexible Parametric Survival Models, Using Income as a Proxy for SES eFigure 6. Estimated Hazard Ratios From Univariable and Multivariable Cox Regressions for SES Differences, Using Income as a Proxy for SES eFigure 7. 2-Year Survival Using 1 and 6 Degrees of Freedom (Spline Knots) in Modeling of the Baseline Hazard eTable. Patient Characteristics by SES, Using Income as a Proxy for SES [file jamanetwopen-e241112-s001.pdf]

Supplemental Online Content

Nielsen LHE, Kristensen DT, Jakobsen LH, et al. Socioeconomic status and overall survival among patients with hematological malignant neoplasms in Denmark. JAMA Netw Open. 2024;7(3):e241112. doi:10.1001/jamanetworkopen.2024.1112

eMethods. Definitions of Variables Used in the Analysis

eFigure 1. All Periods and Unstratified Kaplan-Meier Curves for Overall Survival

eFigure 2. Estimated Hazard Ratios From Univariable and Multivariable Cox Regressions for Socioeconomic Differences: Follow-Up Restricted to 6 Years From Diagnosis

eFigure 3. Unstratified Kaplan-Meier Curves for Overall Survival, Using Income as a Proxy for SES

eFigure 4. Overall Survival Probability by SES and Year of Diagnosis, Using Income as a Proxy for SES

eFigure 5. Estimated SES Differences Using Flexible Parametric Survival Models, Using Income as a Proxy for SES

eFigure 6. Estimated Hazard Ratios From Univariable and Multivariable Cox Regressions for SES Differences, Using Income as a Proxy for SES

eFigure 7. 2-Year Survival Using 1 and 6 Degrees of Freedom (Spline Knots) in Modeling of the Baseline Hazard

eTable. Patient Characteristics by SES, Using Income as a Proxy for SES

This supplemental material has been provided by the authors to give readers additional information about their work.

© 2024 Nielsen LHE et al. *JAMA Network Open.*

eMethods: Definitions of variables used in the analysis

**Definitions**	
Education	Data on highest attained education level were obtained from the Danish Educational registries. As a crude proxy for socioeconomic status, educational level was divided into two groups using Statistics Denmark's classification of education. The high-SES and low-SES group was defined as the education categories:<br>• H40-H80, equivalent to the International Standard Classification of Education 2011 (ISCED-2011) level 4-8<br>• H10-H35, equivalent to ISCED-2011 level 0-3
Income	Data on equivalized family income, a measure of disposable income, were collected from the Danish income registry. Income was calculated as the average of the yearly income 1 to 5 years prior to diagnosis, e.g., if the patient was diagnosed at any time point in 2021, we used the average yearly income from 2016-2020. This number was for each patient dichotomized into a high-SES and low-SES group by whether the income was above or below the median income for the same calendar year and age, given the whole Danish population.
Comorbidity	A modified Charlson comorbidity index (CCI) was calculated based on diagnosis codes given in relation to hospital admissions and medicine administrations by pharmacies. The CCI was constructed without age and cancer diagnoses and was divided into four groups (0, 1, 2, and ≥ 3).
Disease specific prognostic index	Disease-specific prognostic variables were collected from clinical registries. The disease-specific prognostic indices were grouped as follows: MM: ISS (International Staging System for multiple myeloma) as favorable (stage I), intermediate (stage II), and adverse (stage III); AML: Grimwade cytogenetic classification as favorable, intermediate, and adverse; DLBCL: IPI (International Prognostic Index for DLBCL) as favorable (0-1), intermediate (2-3), and adverse (4-5).

© 2024 Nielsen LHE et al. *JAMA Network Open.*

eFigure 1: All periods and unstratified Kaplan-Meier curves for overall survival

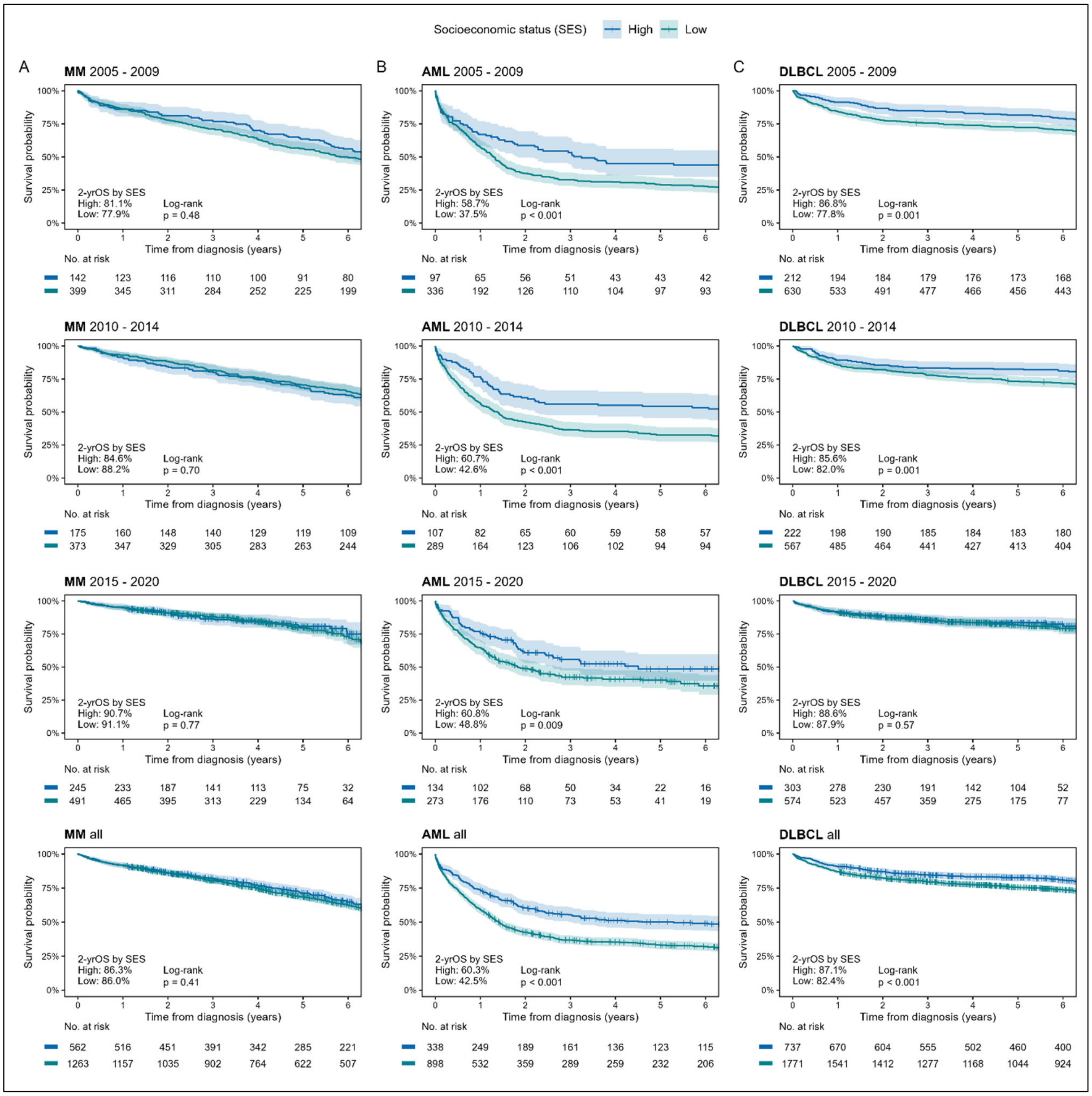

A) MM, multiple myeloma, **B)** AML, acute myeloid leukemia and, **C)** DLBCL, diffuse large B-cell lymphoma.

Abbreviations: OS, Overall survival; SES, socioeconomic status. P-values from Log-rank tests. Censoring marked by vertical line.

© 2024 Nielsen LHE et al. *JAMA Network Open.*

eFigure 2: Estimated hazard ratios from uni- and multivariable Cox regressions for socioeconomic differences (follow-up restricted to 6 years from diagnosis)

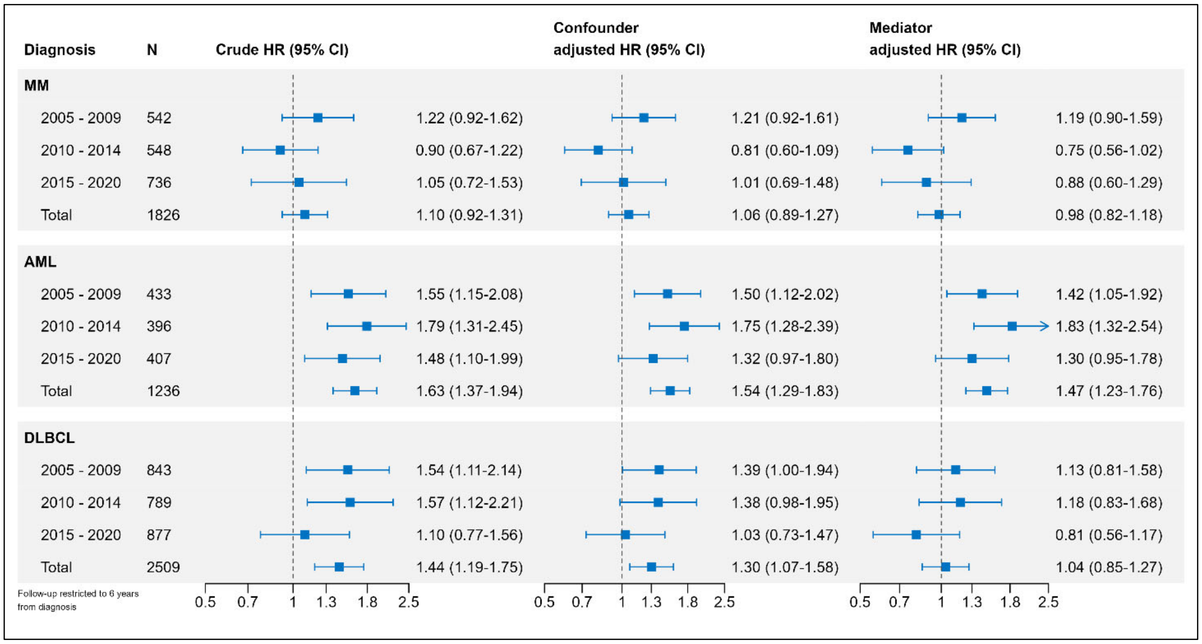

Abbreviations: MM, multiple myeloma; AML, acute myeloid leukemia; DLBCL, diffuse large B-cell lymphoma; HR, hazard ratio; CI, confidence interval.

HR is estimated using high-SES patients (completion of tertiary education) as reference. The confounder-adjusted model includes age and sex, the mediator-adjusted model includes age, sex, performance score, comorbidity, and disease specific prognostic index.

eFigure 3: Unstratified Kaplan-Meier curves for overall survival (using income as a proxy for SES)

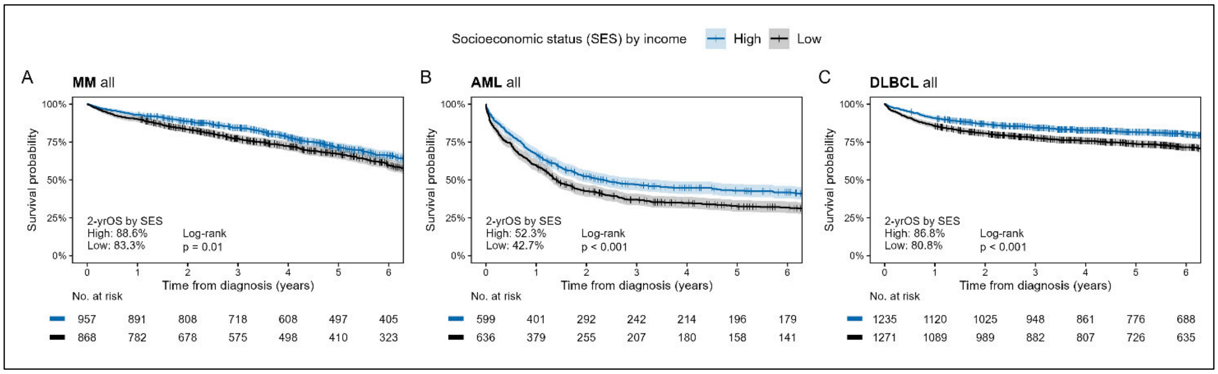

A) MM, multiple myeloma, **B)** AML, acute myeloid leukemia, and **C)** DLBCL, diffuse large B-cell lymphoma.

Abbreviations: OS, Overall survival; SES, socioeconomic status. P-values from Log-rank tests. Censoring marked by vertical line.

© 2024 Nielsen LHE et al. *JAMA Network Open.*

eFigure 4: Overall survival probability by socioeconomic status and year of diagnosis (using income as a proxy for SES)

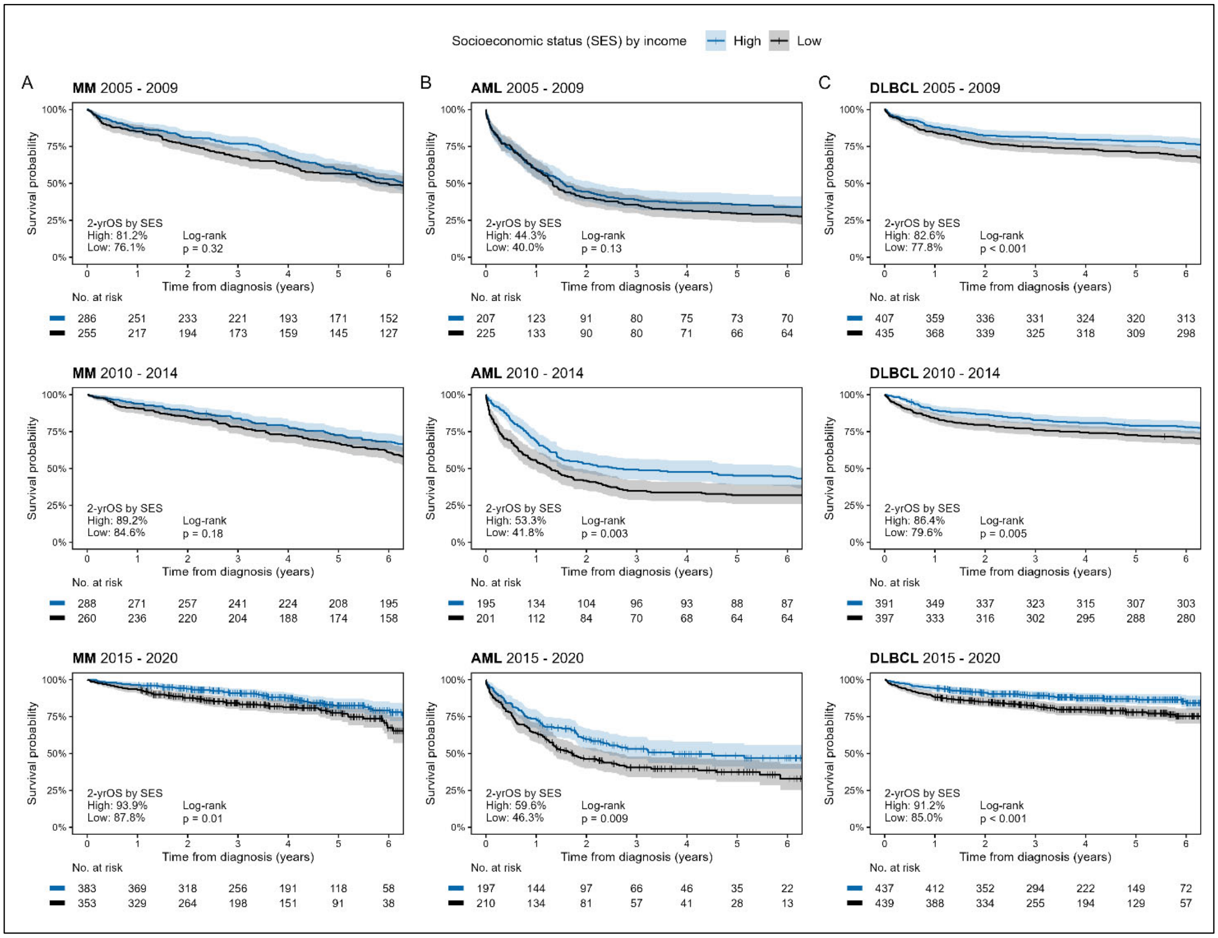

A) MM, multiple myeloma, **B)** AML, acute myeloid leukemia and, **C)** DLBCL, diffuse large B-cell lymphoma. P-values from Log-rank tests. Censoring marked by vertical line.

© 2024 Nielsen LHE et al. *JAMA Network Open.*

eFigure 5: Estimated socioeconomic differences using flexible parametric survival models (using income as a proxy for SES)

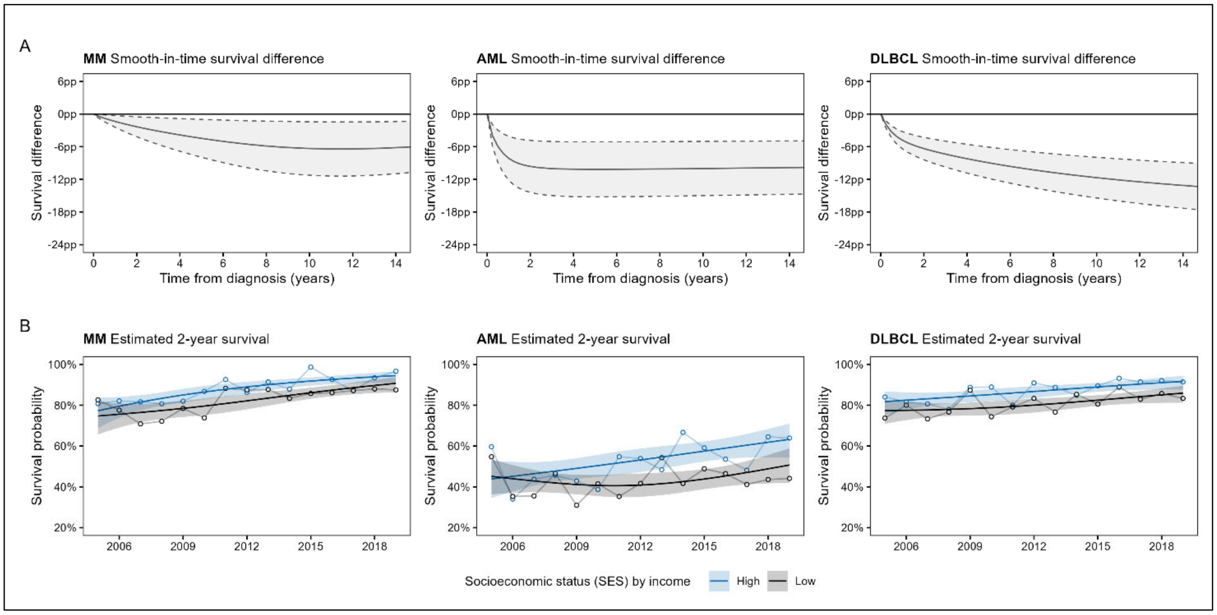

A) Estimated survival difference using flexible parametric survival models 2005 – 2022. **B)** Percentage of 2-year survival through time, raw (points) and smoothed using predictions from a flexible parametric survival model for patients diagnosed from 2005 – 2019 (allows 2-year follow-up).

Abbreviations: MM, multiple myeloma; AML, acute myeloid leukemia; DLBCL, diffuse large B-cell lymphoma; pp, percentage point.

© 2024 Nielsen LHE et al. *JAMA Network Open.*

eFigure 6: Estimated hazard ratios from uni- and multivariable Cox regressions for socioeconomic differences (using income as a proxy for SES)

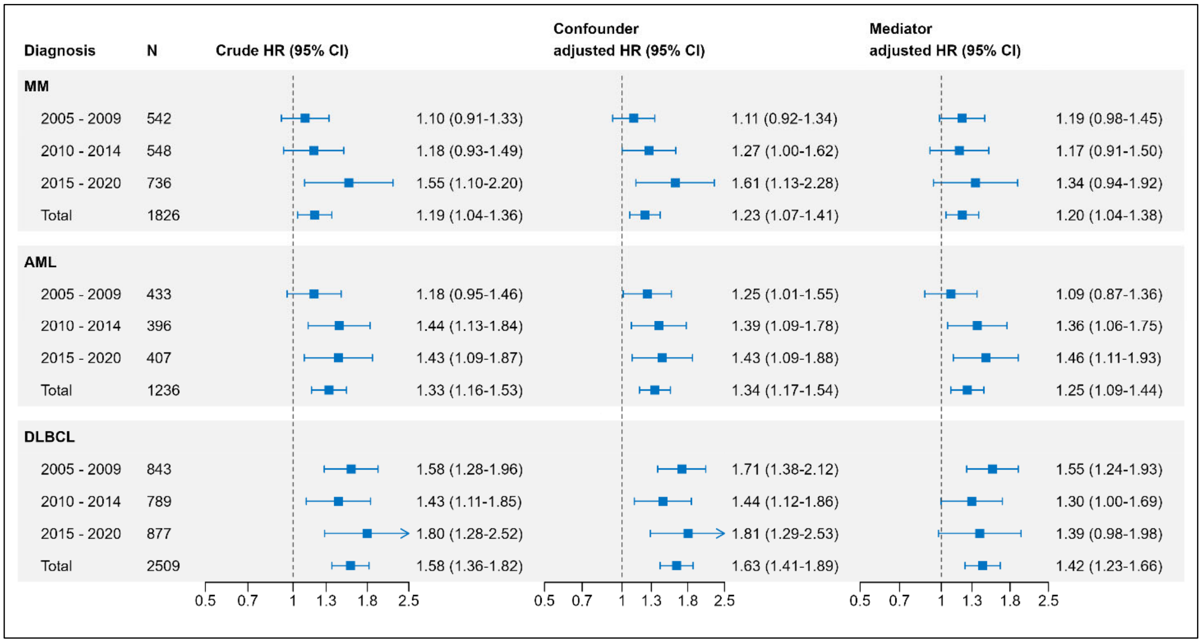

Abbreviations: MM, multiple myeloma; AML, acute myeloid leukemia; DLBCL, diffuse large B-cell lymphoma; HR, hazard ratio; CI, confidence interval.

HR are estimated using high-SES patients (completion of tertiary education) as reference. The confounder-adjusted model includes age and sex, the mediator-adjusted model includes age, sex, performance score, comorbidity, and disease specific prognostic index.

eFigure 7: 2-year survival using 1 and 6 degrees of freedom (spline knots) in modeling of the baseline hazard

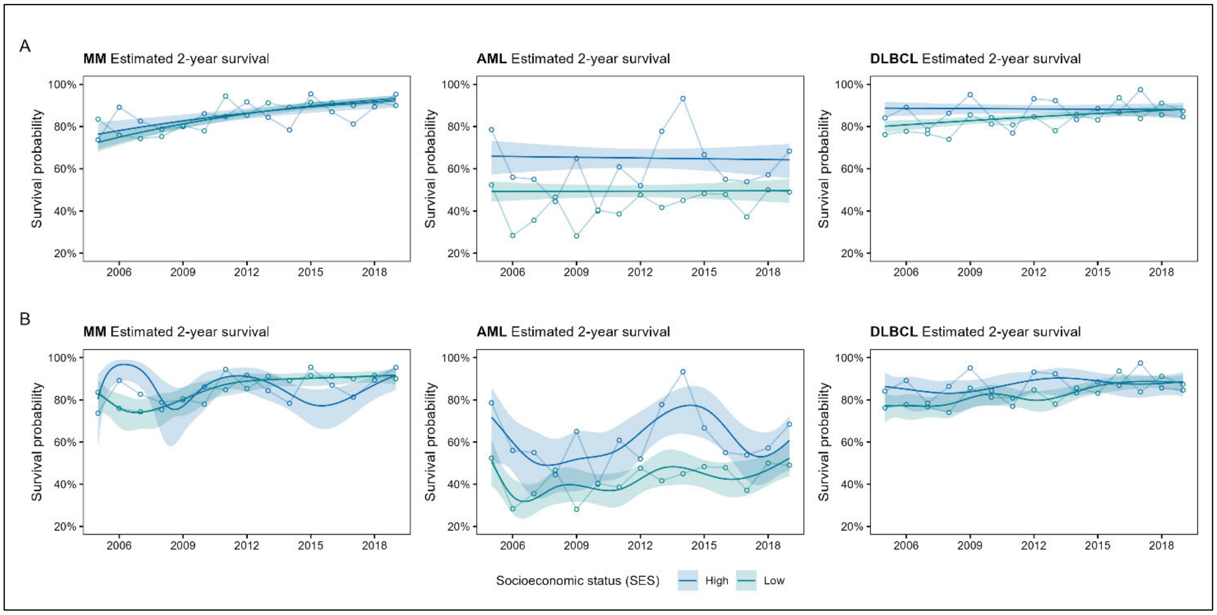

Percentage of 2-year survival through time, discrete (points) and smoothed using a flexible parametric survival model for patients diagnosed from 2005 – 2019 (allows 2-year follow-up) for **A)** 1 degree of freedom, **B)** 6 degrees of freedom.

© 2024 Nielsen LHE et al. *JAMA Network Open.*

eTable 1: Patient characteristics by socioeconomic status (using income as a proxy for SES)

	MM			AML			DLBCL		
**Characteristic**	**Low-SES (n=868)**	**High-SES (n=958)**	**P**	**Low-SES (n=636)**	**High-SES (n=599)**	**P**	**Low-SES (n=1272)**	**High-SES (n=1235)**	**P**
Sex No. (%)			0.23			0.38			0.04
Female	391 (45.0)	403 (42.1)		312 (49.1)	278 (46.4)		538 (42.3)	471 (38.1)	
Male	477 (55.0)	555 (57.9)		324 (50.9)	321 (53.6)		734 (57.7)	764 (61.9)	
Age median (IQR)	58 (53-62)	59 (54-63)		57 (47-62)	56 (47-62)		57 (49-62)	57 (49-62)	
Age group No. (%)			0.29			0.86			0.05
25-39 years	17 (2.0)	19 (2.0)		80 (12.6)	81 (13.5)		107 (8.4)	135 (10.9)	
40-54 years	250 (28.8)	243 (25.4)		195 (30.7)	178 (29.7)		410 (32.2)	368 (29.8)	
55-65 years	601 (69.2)	696 (72.7)		361 (56.8)	340 (56.8)		755 (59.4)	732 (59.3)	
WHO-PS No. (%)			<0.001			<0.001			<0.001
0	346 (39.9)	471 (49.2)		259 (40.7)	306 (51.1)		687 (54.0)	792 (64.1)	
1	319 (36.8)	290 (30.3)		257 (40.4)	226 (37.7)		377 (29.6)	303 (24.5)	
≥ 2	198 (22.8)	185 (19.3)		120 (18.9)	67 (11.2)		204 (16.0)	137 (11.1)	
Immigration No. (%)			<0.001			<0.001			<0.001
Danish	753 (86.8)	922 (96.2)		563 (88.5)	576 (96.2)		1098 (86.3)	1175 (95.1)	
Non-Danish	115 (13.2)	36 (3.8)		73 (11.5)	23 (3.8)		174 (13.7)	60 (4.9)	
Disease-specific prognostic index[a] No. (%)			0.66			0.85			0.76
Favorable	287 (33.1)	325 (33.9)		47 (7.4)	38 (6.3)		518 (40.7)	513 (41.5)	
Intermediate	272 (31.3)	289 (30.2)		309 (48.6)	286 (47.7)		566 (44.5)	558 (45.2)	
Adverse	104 (12.0)	130 (13.6)		154 (24.2)	152 (25.4)		53 (4.2)	48 (3.9)	
Missing	205 (23.6)	214 (22.3)		126 (19.8)	123 (20.5)		135 (10.6)	116 (9.4)	
Charlson comorbidity index No. (%)			<0.001			<0.001			<0.001
0	517 (59.6)	672 (70.1)		418 (65.7)	469 (78.3)		768 (60.4)	888 (71.9)	
1	184 (21.2)	149 (15.6)		146 (23.0)	91 (15.2)		281 (22.1)	234 (18.9)	
2	101 (11.6)	96 (10.0)		31 (4.9)	25 (4.2)		108 (8.5)	47 (3.8)	
≥ 3	66 (7.6)	41 (4.3)		41 (6.4)	14 (2.3)		115 (9.0)	66 (5.3)	
Time period No. (%)			0.91			0.93			0.79
2005 - 2009	255 (29.4)	287 (30.0)		225 (35.4)	207 (34.6)		436 (34.3)	407 (33.0)	
2010 - 2014	260 (30.0)	288 (30.1)		201 (31.6)	195 (32.6)		397 (31.2)	391 (31.7)	
2015 - 2020	353 (40.7)	383 (40.0)		210 (33.0)	197 (32.9)		439 (34.5)	437 (35.4)	

Abbreviations: IQR, interquartile range; SES, socioeconomic status; WHO-PS, WHO performance score; MM, multiple myeloma; AML, acute myeloid leukemia; DLBCL, diffuse large B-cell lymphoma. P-values calculated from Chi-squared test between high-SES group and low-SES group.

[a] Disease-specific prognostic index were categorized as follows. AML: Cytogenetic risk category (Grimwade et al., 2009), Favorable (see ref), Intermediate (see ref), Adverse (see ref). DLBCL: IPI (International Prognostic Index for DLBCL), favorable (0-1), intermediate (2-3), Adverse (4-5). MM: ISS (International Staging System for multiple myeloma), Favorable (stage I), Intermediate (stage II), Adverse (stage III).

© 2024 Nielsen LHE et al. *JAMA Network Open.*
