# Supplementary material for: Socioeconomic Status and Overall Survival Among Patients With Hematological Malignant Neoplasms
Source: JAMA Netw Open. 2024 Mar 4;7(3):e241112. doi: 10.1001/jamanetworkopen.2024.1112 (PMC10912957; doi:10.1001/jamanetworkopen.2024.1112)
Supplement: Supplement 2. — Data Sharing Statement [file jamanetwopen-e241112-s002.pdf]

Data Sharing Statement

Nielsen. Socioeconomic Status and Overall Survival Among Patients With Hematological Malignant Neoplasms in Denmark. *JAMA Netw Open*. Published March 04, 2024. doi:10.1001/jamanetworkopen.2024.1112

Data
Data available: No

Additional Information
Explanation for why data not available: The participants of this study did not give consent for their data to be shared publicly, so due to the sensitive nature of the research supporting data is not available.
